# Applications of the Conceptual Density Functional Theory Indices to Organic Chemistry Reactivity

**DOI:** 10.3390/molecules21060748

**Published:** 2016-06-09

**Authors:** Luis R. Domingo, Mar Ríos-Gutiérrez, Patricia Pérez

**Affiliations:** 1 Department of Organic Chemistry, University of Valencia, Dr. Moliner 50, E-46100 Burjassot, Valencia, Spain; M.Mar.Rios@uv.es; 2 Facultad de Ciencias Exactas, Departamento de Ciencias Químicas, Universidad Andres Bello, Av. República 498, 8370146 Santiago, Chile; p.perez@unab.cl

**Keywords:** conceptual DFT, reactivity indices, molecular electron density theory, electrophilicity, nucleophilicity, Parr functions

## Abstract

Theoretical reactivity indices based on the conceptual Density Functional Theory (DFT) have become a powerful tool for the semiquantitative study of organic reactivity. A large number of reactivity indices have been proposed in the literature. Herein, global quantities like the electronic chemical potential μ, the electrophilicity ω and the nucleophilicity *N* indices, and local condensed indices like the electrophilic $P_{k}^{+}$ and nucleophilic $P_{k}^{-}$ Parr functions, as the most relevant indices for the study of organic reactivity, are discussed.

## 1. Introduction

Since the introduction of the chemical bond concept by Lewis at the beginning of the 20th century [1], two quantum-chemical theories, namely, the Valence Bond (VB) theory [2,3,4] and the Molecular Orbital (MO) theory [5] based on Schrödinger’s equation [6], have been developed. In the 60s of the last century, based on the Hohenberg and Kohn theorems, a new quantum-chemical theory to study the structure of matter, known as the Density Functional Theory (DFT) [7], in which the ground state energy of a non-degenerate N-electron system is a unique functional of the density ρ(**r**), was established:

(1) E[ρ(r)] = ∫ρ(r)v(r) dr + F[ρ(r)]

F[ρ(**r**)] is the universal functional of Hohenberg-Kohn given by the sum of the kinetic energy functional, T[ρ(**r**)], and the electron–electron interaction energy functional, V_ee_[ρ(**r**)], and *v*(**r**) is the “external one electron potential”, *i.e.*, the electron nucleus Coulomb interaction. This theorem constitutes the rigorous theoretical foundation of the DFT. Within the DFT framework, the electron density can be expressed as the functional derivative of the energy with respect to the external potential, the number of electrons being kept constant:

(2) $$\rho \left(r\right)={\left(\frac{\delta E}{\delta v\left(r\right)}\right)}_{N}$$

Thus, DFT calculations imply the construction of an expression of the electron density. Similar to the quantum-chemical theory based on Schrödinger’s equation, the resolution of the functional of the electron density ρ(**r**) for a complex system neither is computationally feasible. The mathematical problem is the definition of each term of the functional F[ρ]. As an approximation, the Kohn-Sham formalism [8] was introduced in analogy to the Hartree-Fock equation. In the last decades, a series of empiric DFT functionals, such as B3LYP [9,10], MPWB1K [11] and, more recently, M06 and related functionals [12], which provide accurate energies, have been developed, making the study of organic reactions with a computational demand similar to MO calculations possible.

The development of the topological analysis of the Electron Localisation Function [13] (ELF) at the end of the 20th century allowed understanding a rigorous quantum-chemical analysis of the molecular electron density in terms of non-bonding and bonding molecular regions. This analysis makes the creation of a molecular picture that can be related to the Lewis bonding pattern possible.

The ELF topological analysis of the bonding changes along a reaction path has allowed establishing the molecular mechanism of most organic reactions. Numerous studies of organic reactions involving C–C bond formation processes have enabled the establishment of a reactivity model in which these bonds are formed through the C-to-C coupling of two *pseudoradical* centers [14] generated along the reaction path [15]. Interestingly, non-polar, polar and ionic reactions involving C–C double bonds show this pattern. In non-polar reactions, the energy demanded for the rupture of the C–C double bonds, which is required to reach the *pseudoradical* structures, accounts for the high activation energies associated with non-polar processes. It is noteworthy that these high activation energies decrease with the increase of the polar character of the reaction. In this sense, Domingo proved that the Global Electron Density Transfer [15] (GEDT) taking place in polar processes favours the changes in electron density demanded for the C–C single bond formations.

These findings have recently made it possible to establish the Molecular Electron Density Theory [16] (MEDT), which states that “*while the electron density distribution at the ground state is responsible for physical and chemical molecular properties, as proposed DFT [7], the capability for changes in electron density, and not molecular orbital interactions, is responsible for molecular reactivity*” [15]. Indeed, it was Parr who established that the electron density distribution at the ground state is responsible for the chemical properties of molecules and, accordingly, MEDT proposes that the reactivity in organic chemistry should be studied analysing the electron density reorganisation along a chemical reaction.

Within DFT, a series of chemical molecular properties derived from the exchange of electron density at the ground state of the molecules have been developed, becoming powerful tools for an earlier study of the molecular reactivity in polar processes, which are characterised by the flux of GEDT from electron donor molecules (nucleophiles) to electron acceptor molecules (electrophiles).

## 2. Conceptual DFT

One of the most relevant traits of the density functional language is its suitability for defining and elucidating important chemical concepts of molecular structure and reactivity. Parallel to the development of quantum-chemical models to approach the Hohenberg-Kohn equation [7], Parr developed the so-called “conceptual DFT” in the late 1970s and early 1980s [17]. Conceptual DFT is a DFT-subfield in which one tries to extract from the electron density relevant concepts and principles that make it possible to understand and predict the chemical behaviour of a molecule. Parr and co-workers, and later a large community of theoretical chemists, have been able to give precise definitions for chemical concepts which had already been known and used for many years in various branches of chemistry, electronegativity as the most noticeable example, thus providing their calculations with a quantitative use. Herein, the most relevant indices defined within the conceptual DFT [18] for the study of the organic reactivity are discussed.

Conceptual DFT essentially relies on the fact that the ground state energy of an N-electron system as given by the Hohenberg-Kohn theorem (Equation (1)) can be considered as depending upon the number of electrons N and the external potential *v*(**r**), which are themselves determined solely by the density, in other words E[ρ(**r**)] = E[N;*v*(**r**)]. In this context, the responses of the system to changes of the number of its electrons, of the external potential or of both, provide information about its reactivity.

**Figure 1:**
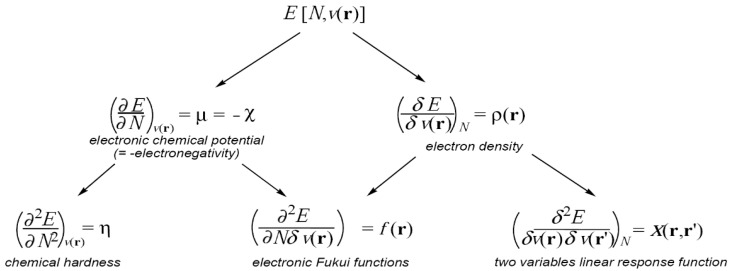
First and second derivatives of E[N;*v*(**r**)] with respect to N and *v*(**r**).

The E[N;*v*(**r**)] derivatives with respect to N and *v*(**r**) constitute a first series of reactivity indicators, the electronic chemical potential μ, which is the opposite of the electronegativity χ, the chemical hardness η, the Fukui function *f*(**r**) and the two variables linear response function χ(**r**,**r′**), as shown in the diagram given in Figure 1. The left side properties in the diagram are global properties, *i.e.*, their values are the same wherever the position they are calculated, whereas the right side ones are local functions of one or two coordinate variables, *i.e.*, their values depend on the position where they are evaluated.

Herein, the electronic chemical potential μ, the electronegativity χ, the chemical hardness η, the electrophilicity ω and nucleophilicity *N* indices, and the Fukui *f*(**r**) and Parr *P*(**r**) functions, as well as their applications in the study of organic chemical reactivity, will be discussed. Non-local response functions such as *X*(**r**,**r′**) are out of the scope of this review.

### 2.1. Electronic Chemical Potential μ and Mulliken Electronegativity χ

In 1983, Parr defined the electronic chemical potential μ as the energy changes of the system with respect to the electron number N at a fixed external potential *v*(***r***), *i.e.*, the potential created by the nuclei (see Equation (3)) [19,20]. The electronic chemical potential μ is associated with the feasibility of a system to exchange electron density with the environment at the ground state.

(3) $$µ\;=\;{\left(\frac{\partial E}{\partial N}\right)}_{v\left(r\right)}$$

Applying the finite difference approximation, the following simple expression is obtained:

(4) $$µ\;\approx \;-\;\frac{\left(\text{I }+\text{ A}\right)}{2}$$

where I and A are the ionisation potential and the electron affinity of an atom or molecule, respectively. Although a large number of experimental I values for organic molecules can be obtained, a very small number of experimental A values can be found. Using Koopmans theorem [21] and Kohn–Sham formalism [8] within the DFT, these energies can be approached by the frontier HOMO and LUMO energies as I by −$E_{HOMO}$ and A by −$E_{LUMO}$. Consequently, the electronic chemical potential μ can be expressed as:

(5) $$µ\;\approx \;\frac{\left(E_{HOMO}+\;E_{LUMO}\right)}{2}$$

The electronic chemical potential μ of some reagents involved in Diels-Alder reactions is given in Table 1. In this short series, while reagents such as tetracyanoethylene **1a**, μ = −7.04 eV, located at the top of Table 1, will act as strong electron-acceptor molecules, reagents located at the bottom such as dimethylvinyl amine **1j**, μ = −1.85 eV, will act as strong electron-donor molecules. As can be seen, butadiene **1f**, μ = −3.46 eV, and cyclopentadiene **1h**, μ = −3.01 eV, present a similar electronic chemical potential μ as ethylene **1g**, μ = −3.37 eV. Consequently, it is expected that the Diels-Alder reactions of dienes **1f** or **1h** with ethylene **1g** will present a low polar character [22]. The increase of the electron-withdrawing character of the substituent present in ethylene **1g**, –CHO < –CN < –NO_2_, decreases the electronic chemical potential μ of the corresponding ethylene derivative, **1e** < **1d** < **1c**, thus increasing the polar character of the reactions towards dienes **1f** or **1h**.

The identification of the electronic chemical potential μ with the negative of Mulliken electronegativity, −χ, which is a measure of the resistance to electron density loss, offers a way to calculate electronegativity values for atoms and molecules. In this sense, it was an important step forward, as there was not a systematic way to evaluate electronegativities for atoms and molecules with the existing scales established by Pauling [23,24].

(6) $$\chi \;=\;-µ\;\approx \;\frac{\left(\text{I }+\text{ A}\right)}{2}$$

#### Electronegativity Equalisation Principle

According to the electronegativity equalisation principle, primarily formulated by Sanderson [25,26,27,28], “when two or more atoms initially different in electronegativity combine chemically, their electronegativities become equalised in the molecule”. The correctness of Sanderson’s principle immediately comes from the fact that the electronic chemical potential μ is a property of an equilibrium state. Consequently, when two atoms A and B, with ${µ}_{A}$ < ${µ}_{B}$, approach one another, there is a flux of electron density from B, the less electronegative atom, towards A, the more electronegative one, to equilibrate the electronic chemical potential ${µ}_{AB\;}$in the new interacting system.

Accordingly, the electronic chemical potential μ allows the establishment of the flux direction of the GEDT [15] along a polar reaction. Likewise, in a polar reaction involving two molecules, A and B, with ${µ}_{A}$ < ${µ}_{B}$, the electron density flux will take place from molecule B, which has the higher μ, towards molecule A, which has the lower μ. Therefore, along a polar process, while A will act as the electron-acceptor, *i.e.*, the electrophile, B will act as the electron-donor, *i.e.*, the nucleophile. The larger the electronic chemical potential difference, Δμ, the larger the GEDT.

### 2.2. Chemical Hardness η and Softness S

In 1963, Pearson established a classification of Lewis acids and bases into hard and soft [29,30,31]. He proposed that in an acid/base reaction, the most favourable interactions take place between hard/hard or soft/soft pairs, the hard and soft acids and bases (HSAB) principle. Within the conceptual DFT, Parr defined, in 1983, a quantitative expression for the chemical hardness η, which can be expressed as the changes of the electronic chemical potential μ of the system with respect to the electron number N at a fixed external potential *v*(**r**) [19]:

(7) $$\eta \;=\;{\left(\frac{\partial µ}{\partial N}\right)}_{v\left(r\right)}=\;{\left(\frac{\partial^{2}E}{\partial N^{2}}\right)}_{v\left(r\right)}$$

The chemical hardness η can be thought as a resistance of a molecule to exchange electron density with the environment.

Applying the finite difference approximation, the following simple expression is obtained:

(8) $$\eta \;\approx \;\frac{\left(I\;-\;A\right)}{2}$$

which by substitution of I by −$E_{HOMO}$ and A by −$E_{LUMO}$ can be expressed as:

(9) $$\eta \;\approx \;\frac{\left(E_{LUMO}\;-\;E_{HOMO}\right)}{2}$$

Usually, the term 1/2 is neglected, so that the chemical hardness η is expressed as:

(10) $$\eta \;\approx \;(E_{LUMO}\;-\;E_{HOMO})$$

On the other hand, the chemical softness S was introduced as the inverse of the chemical hardness η:

(11) $$S\;=\;\frac{1}{\eta }$$

#### The HSAB Principle and the Maximum Hardness Principle (MHP)

On the basis of a variety of experimental data, Pearson [29,30] presented a classification of Lewis acids and bases and later provided the HSAB principle [31,32,33], which states that “among the potential partners of same electronegativity, hard likes hard and soft likes soft”. On the other hand, it was established that there exists a relationship between energy and hardness variations [19]. This relationship is based on the MHP that establishes that, in any chemical process, the systems evolve toward an electronic state where its chemical hardness is a maximum [19]. When combined with the minimum energy criterion that characterises stability, the MHP entails that, for a chemical process characterised by a negative (stabilising) energy change, the change in chemical hardness will always be positive.

### 2.3. The Fukui Functions f(**r**)

In 1984, in a short communication entitled “*Density Functional Approach to the Frontier-Electron Theory of Chemical Reactivity*”, Parr [34] proposed the *f*(**r**) function, named frontier function or Fukui function, for a molecule, which was defined as:

(12) $$f(r)\;=\;{\left(\frac{\partial \rho \left(r\right)}{\partial N}\right)}_{v\left(r\right)}$$

The Fukui function *f*(**r**) represents the changes in electron density at a point **r** with respect to the variation of the number of electrons N at a fixed external potential *v*(**r**).

Parr assumed that the preferred direction for the approach of a reagent towards the other is the one for which the initial variation of the electronic chemical potential μ for a species is a maximum, and the one with the largest *f*(**r**) is situated at the reaction site [34]. Using the frozen core approximation, in which δρ = δ$\rho_{VALENCE}$, Parr proposed:

(13) $$f^{-}(r)\;\approx \;\rho_{HOMO}(r)\;\;\;\mathrm{for}\;\mathrm{electrophilic}\;\mathrm{attacks}$$

and

(14) $$f^{+}(r)\;\approx \;\rho_{LUMO}(r)\;\;\;\mathrm{for}\;\mathrm{nucleophilic}\;\mathrm{attacks}$$

Although, in principle, the electron density of a neutral or N_0±1electron_ molecule contains all the information needed for the evaluation of the Fukui function *f*(**r**), most studies in the literature have been carried out in the so-called finite difference method, in which the Fukui functions *f*(**r**) are approximated as [35]:

(15) $$f^{-}(r)\;\approx \;\rho_{No}(r)\;-\;\rho_{N-1}(r)\;\;\;\mathrm{for}\;\mathrm{electrophilic}\;\mathrm{attacks}$$

and

(16) $$f^{+}(r)\;\approx \;\rho_{N+1}(r)\;-\;\rho_{No}(r)\;\;\;\mathrm{for}\;\mathrm{nucleophilic}\;\mathrm{attacks}$$

where $\rho_{No}$, $\rho_{No-1}$and $\rho_{No+1}$are the atomic charges in the neutral, cationic and anionic species.

### 2.4. The Electrophilicity ω Index

In 1999, Parr defined the electrophilicity ω index [36], which gives a measure of the energy stabilisation of a molecule when it acquires an additional amount of electron density, ΔN, from the environment. The electrophilicity ω index is given by the simple expression:

(17) $$\omega \;=\;\frac{{µ}^{2}}{2\eta }$$

The electrophilicity ω index encompasses the tendency of an electrophile to acquire an extra amount of electron density, given by μ, and the resistance of a molecule to exchange electron density with the environment, given by η. Thus, a good electrophile is a species characterised by a high |μ| value and a low η value.

Besides, the maximum number of electrons that an electrophile can acquire is given by the expression [36]:

(18) $$∆N_{max}=\;-\frac{\text{µ}}{\eta }$$

The electrophilicity ω index has become a powerful tool for the study of the reactivity of organic molecules participating in polar reactions [37]. A comprehensive study carried out in 2002 on the electrophilicity of a series of reagents involved in Diels-Alder reactions allowed establishing a single electrophilicity ω scale (see Table 2) [38]. In 2003, the three-atom-components (TACs) participating in [3+2] cycloaddition (32CA) reactions were studied using the electrophilicity ω index, allowing a rationalisation of their reactivity in polar processes [39]. The electrophilicity ω scale allowed the classification of organic molecules as strong electrophiles with ω > 1.5 eV, moderate electrophiles with 0.8 < ω < 1.5 eV and marginal electrophiles with ω < 0.8 eV [38].

Strong electrophiles such as 1,1-dicyanoethylene **2d**, ω = 2.82 eV, and nitroethylene **2e**, ω = 2.61 eV, presenting an electrophilicity ω index higher than 2.0 eV, participate easily in polar Diels-Alder reactions [40]. Ethylene **2n**, ω = 0.73 eV, is a marginal electrophile that is not able to participate in polar processes. Substitution of a hydrogen atom of ethylene **2n** by an electron-withdrawing carbonyl group increases the electrophilicity ω index of acrolein **2f** to 1.84 eV. Although acrolein is classified as a strong electrophile, it needs an additional electrophilic activation to participate in polar processes under mild conditions. Thus, even the coordination of a weak Lewis acid such as BH_3_ to the carbonyl oxygen atom of acrolein **2f** notably increases the electrophilicity ω index of the acrolein:BH_3_ complex **2c** to 3.20 eV. This electrophilic activation of acrolein in the complex accounts for the role of Lewis acid catalysts in organic reactions favouring the reactions to take place *via* a more polar process [40]. Cationic species such as *N*-dimethylmethyleneammonium cation **2a**, ω = 8.25 eV, are the most electrophilic organic species, participating in ionic organic reactions [41].

Species such as ethylene **2n**, ω = 0.73 eV, methyl vinyl ether **2q**, ω = 0.42 eV or dimethylvinylamine **2r**, ω = 0.27 eV, with an electrophilicity ω index below 0.80 eV, are classified as marginal electrophiles. Interestingly, diene species such as *N*,*N*′-dimethyl-1,3-buta-1,3-dien-1-amine **2o**, or ethylenes such as *N*,*N*′-dimethyl-vinylamine **2r**, participate in polar reactions as good nucleophiles. Consequently, for the short series of simple marginal electrophiles given in Table 2, a good correlation between the inverse of the electrophilicity ω index and the nucleophilicity was established; for these marginal electrophilic species, the less electrophilic they are, the more nucleophilic they are.

**Scheme 1:**
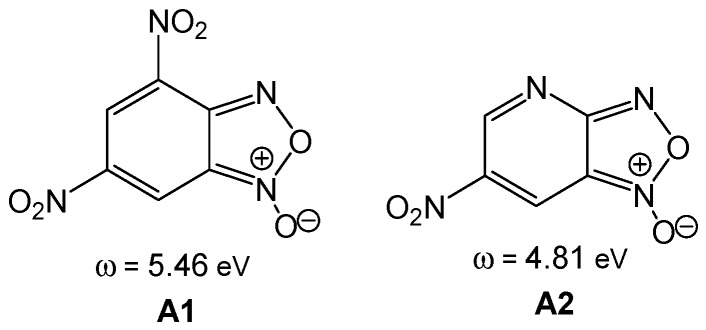
Nitrobenzofuroxans **A1** and **A2**.

Nitrobenzofuroxans such as 4,6-dinitrobenzofuroxan **A1** and 4-aza-6-nitrobenzofuroxan **A2** (see Scheme 1) are compounds with a high susceptibility to undergo nucleophilic addition or substitution processes with very weak nucleophiles [42]. Terrier studied the high reactivity of these species, classifying them as *superelectrophiles* [43]. Thus, neutral species such as 4,6-dinitrobenzofuroxan **A1**, ω = 5.56 eV, and 4-aza-6-nitrobenzofuroxan **A2**, ω = 4.81 eV, presenting an electrophilicity ω index higher than 4.00 eV, are *superelectrophiles* showing high reactivity in polar reactions [44]. Note that *superelectrophilic* species do not react in ionic reactions; the ionic character of an organic reaction is determined by the participation of cationic or anionic species throughout the reaction [41].

**Figure 2:**
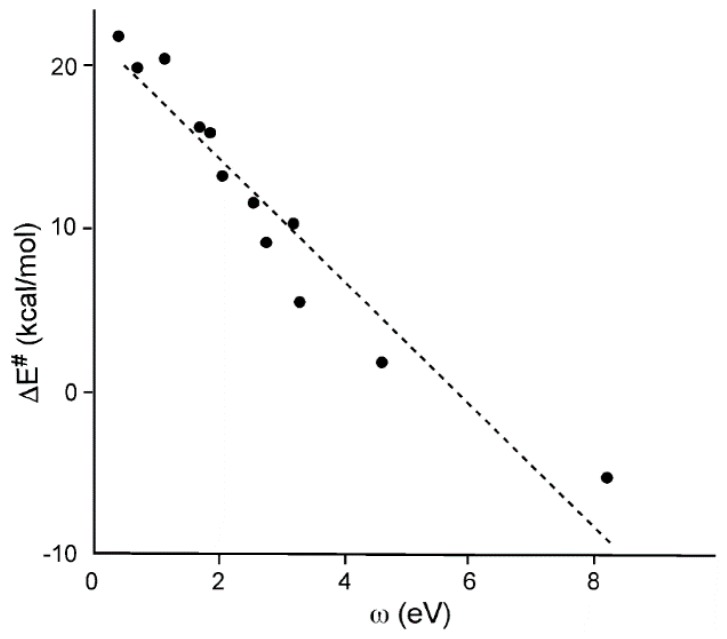
Plot of the activation barriers, ΔE≠, *versus* the electrophilicity ω index of a series of ethylenes involved in Diels-Alder reactions with cyclopentadiene 2l, R^2^ = 0.92 (see reference [40]).

In 2009, Domingo studied the Diels-Alder reactions of cyclopentadiene **2l** with twelve ethylenes of increased electrophilicity [40]. For this short series, a good correlation between the computed activation energies and the electrophilicity ω index of these ethylene derivatives was found (R^2^ = 0.92) (see Figure 2).

As commented, in the short series of simple molecules participating in Diels-Alder reactions given in Table 2, a good correlation between the inverse of the electrophilicity ω index of the marginal electrophiles and its expected nucleophilicity was found. In a polar reaction, the more electrophilic a reagent is and more nucleophilic the other is, the higher the GEDT that usually takes place. This behaviour accelerates polar reactions through more zwitterionic TSs. In this first study involving simple molecules, a good correlation between the difference of the electrophilicity of the two reagents, Δω, and the polar character of the reaction was established. Thus, it is expected that the Diels-Alder reaction between a pair of reagents located at the extreme positions of Table 1 and presenting a Δω higher than 3.00 eV takes place easily via a highly polar reaction.

(19) $$∆\omega \;=\;\omega_{electrophile}\;-\;\omega_{nucleophile}$$

**Scheme 2:**
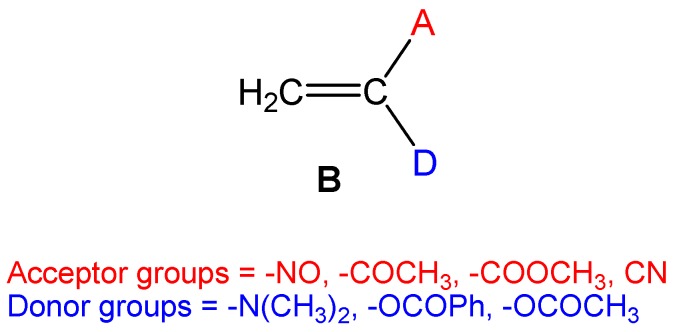
Captodative etylenes B.

However, this prediction fails for more complex molecules such as captodative ethylenes B (see Scheme 2), which display concurrently both electrophilic and nucleophilic behaviours (see later). For these species having two or more functional groups of a different electron demand, the value of the electrophilicity ω index does not correlate well with their expected nucleophilicity.

### 2.5. The Nucleophilicity N Index

While for the electrophilicity ω index only one expression has been established, several approaches for the nucleophilicity index have been gaven. In 1998, Roy [45] proposed the “relative electrophilicity” ($S_{k}^{+}$*^+^*/$S_{k}^{-}$) and the “relative nucleophilicity” ($S_{k}^{-}$/$S_{k}^{+}$) descriptors for a k atom, as a way to locate the preferable electrophilic and nucleophilic reactive sites for the study of the intermolecular reactivity. The electrophilic and nucleophilic local softness $S_{k}^{+}$ and $S_{k}^{-}$ [45] are given by the following equations:

(20) $$S_{k}^{+}\;=\;S\cdot f_{k}^{+}$$

(21) $${S_{k}^{-}}^{-}\;=\;S\cdot f_{k}^{-}$$

where S is the chemical softness and $f_{k}^{+}$ and $f_{k}^{-}$ are the electrophilic and nucleophilic Fukui functions.

In 2003, Chattaraj proposed three generalised philicity $\omega_{k}^{\alpha }$ (α = +, − or o) indices to identify the most electrophilic, nucleophilic and radical sites in reactivity and regioselectivity studies [46]. The condensed (or local) electrophilicity $\omega_{k}^{+}$ and nucleophilicity $\omega_{k}^{-}$ indices were related to the Parr global electrophilicity ω index and the corresponding Fukui functions by Equations (22) and (23).

(22) $$\omega_{k}^{+}\;=\;\omega \cdot f_{k}^{+}$$

(23) $$\omega_{k}^{-}\;=\;\omega \cdot f_{k}^{-}$$

In 2003, Contreras described a method to estimate the relative nucleophilicity for a series of neutral and charged electron-donor species from their solution phase ionisation potential, I_s_ (Equation (24)) [47].

(24) $$N\;=\;-I_{s}$$

**Figure 3:**
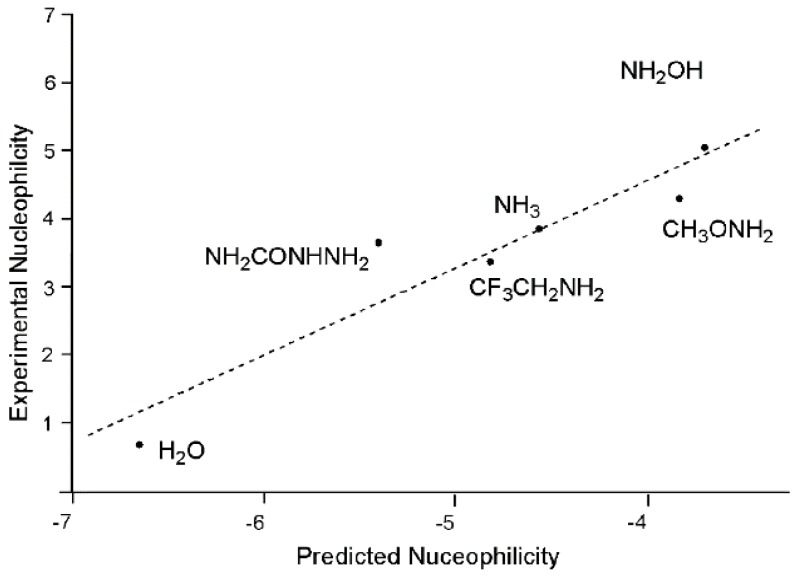
Comparison between Mayr’s experimental nucleophilicity N^+^ and the predicted solution nucleophilicity obtained at the IPCMMP2/6-311G(d,p) level for a series of first-row electron donors.

A very good correlation between Mayr’s experimental nucleophilicity N^+^ [48] and the predicted solution nucleophilicity obtained by Equation (24) at the IPCM-MP2/6-311G(d,p) level for a series of first-row electron donors was found (see Figure 3).

In 2007, Gázquez introduced the concepts of the electroaccepting, ω^+^, and electrodonating, ω^−^, powers as [49]:

(25) $$\omega^{+}=\;\frac{A^{2}}{2\left(I-A\right)}$$

(26) $$\omega^{-}=\;\frac{I^{2}}{2\left(I-A\right)}$$

where ω^+^ represents a measure of the propensity of a given system to accept electron density, while ω^−^ represents the propensity of this system to donate electron density.

In 2008, we proposed an empirical (relative) nucleophilicity *N* index for closed-shell organic molecules based on the HOMO energies, $E_{HOMO}$, obtained within the Kohn-Sham scheme as an approach to the gas phase I, and defined as [50]:

(27) $$N\;=\;E_{HOMO}(\mathrm{Nucleophile})\;-\;E_{HOMO}(\mathrm{TCE})$$

The nucleophilicity *N* index is referred to tetracyanoethylene (TCE) **2b**, which is the most electrophilic neutral species in Table 2, *i.e.*, the expected least nucleophilic neutral species. This choice allowed convenient handling of a nucleophilicity scale of positive values.

An analysis of a series of common nucleophilic species participating in polar organic reactions allowed a further classification of organic molecules as strong nucleophiles with *N* > 3.0 eV, moderate nucleophiles with 2.0 ≤ *N* ≤ 3.0 eV and marginal nucleophiles with *N* < 2.0 eV (see Table 3) [51].

As can be seen, while water, **3z**, *N* = 1.20 eV, is the poorest nucleophile of this series, hydrazine **3c**, *N* = 3.65 eV, is one of the best nucleophilic species. Alkyl substitution on the marginal nucleophilic ethylene **3x** increases its nucleophilicity *N* index from 1.86 eV to 3.35 eV in tetramethylethylene **3f**, being classified as a strong nucleophile. Table 3 also shows that the nucleophilicity *N* index predicts correctly the activation/deactivation of benzene with the substitution. Finally, while ethylene **2n** is a marginal electrophile, ω = 0.73 eV (see Table 2), and a marginal nucleophile, **3x**, *N* = 1.86 eV (see Table 3), 1,3-butadiene **2j** is a moderate electrophile, ω = 1.05 eV (see Table 2), and on the borderline of strong nucleophiles, **3j**, *N* = 2.89 eV (see Table 3). Consequently, while ethylene **3x** cannot participate in polar Diels-Alder reactions, butadiene **3j** participates as a very good nucleophile. These results completely agree with the experimental observation that while the non-polar Diels-Alder reaction between butadiene and ethylene is one of the most unfavourable ones, only the electrophilic activation of ethylene makes the reaction feasible [40].

**Figure 4:**
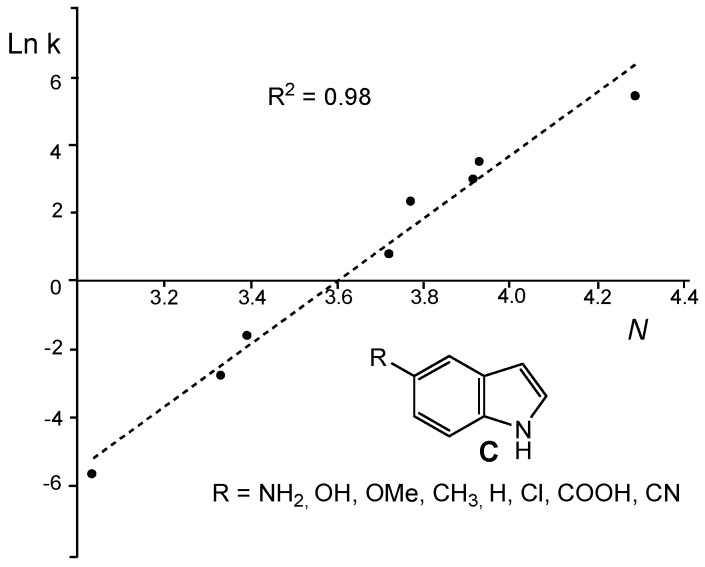
Plot of the experimental Ln k and the calculated gas-phase nucleophilicity *N* index, in eV, for a series of 5-substituted indoles **C** in the reaction with a benzhydryl cation.

Several theoretical and experimental studies have evidenced the capability of the nucleophilicity *N* index to predict the nucleophilic behaviour of organic molecules. Figure 4 shows a good correlation (R^2^ = 0.89) between the experimental Ln k for the reactions of a series of 5-substituted indoles **C** with a benzhydryl cation [52] and the nucleophilicity *N* index of the former [53].

Regarding Chattaraj’s philicity $\omega_{k}^{\alpha }$ indices, a greater $\omega_{k}^{+}$ value corresponds to a better capability of accepting electron density, whereas a smaller value of $\omega_{k}^{-}$ of a system makes it a better donor of electron density. In order to equalise with the general notion that “more is better”, in 2010, Roy proposed the nucleophilicity *N*′ index as the inverse of Gázquez’s electrodonating ω^−^ power [54]. Moreover, since the nucleophilicity *N*′ index obtained as the inverse of ω^−^ was below 1, the authors later defined the nucleophilicity *N*′ index as:

(28) $$N\prime \;=\;\frac{10}{\omega^{-}}$$

To validate the nucleophilicity *N*′ index, the nucleophilicity values of sixty-nine commonly used arenes and heteroarenes were calculated and the corresponding values were compared with the Hammett σ and σ_p_ values [55,56]. In each case, an excellent correlation with the experimental results was obtained. It is interesting to remark that the selected arenes and heteroarenes series are simple molecules having only one functional group, for which, similar to the simple molecules given in Table 1, the inverse of the Parr electrophilicity ω index also gives good correlations [54].

**Scheme 3:**
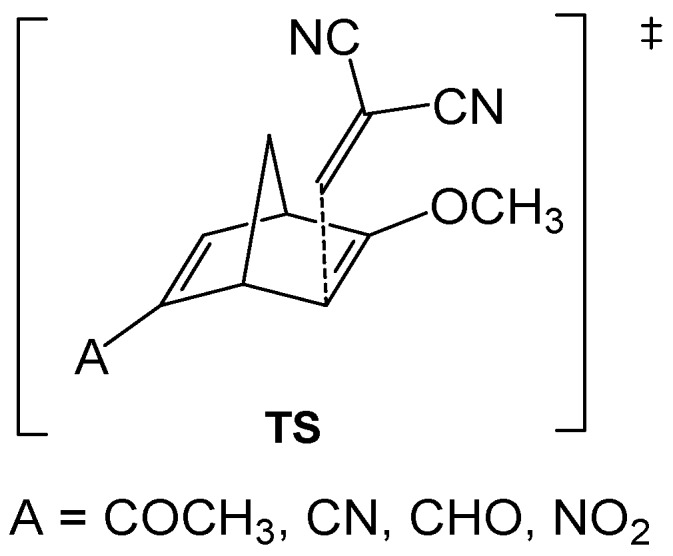
TS associated with the nucleophilic attack of hepta-2,5-dienes **D** on 1,1-dicyanoethylene **E**.

In order to test the nucleophilicity *N* and *N*′ indices, (i) the series of captodative ethylenes **B** given in Scheme 2 and (ii) the series of 2,5-disubstituted bicyclic[2.2.1]hepta-2,5-dienes **D** given in Scheme 3, which display concurrently electrophilic and nucleophilic behaviours, were considered [53].

While the two indices account well for the nucleophilic behaviour of organic molecules having only one substitution, the nucleophilicity *N* index works better for more complex molecules presenting a dual reactivity, but the *N*′ index fails. Thus, captodative ethylene **4b**, having one of the most electron-withdrawing groups, the nitroso –NO one, and one of the most electron-releasing groups, the amino –N(CH_3_)_2_ one, presents a high electrophilicity ω index, 2.52 eV, being classified as a strong electrophile, and a high nucleophilicity *N* index, 3.29 eV, also being classified as a strong nucleophile, in clear agreement with its expected dual reactivity (see Table 4). However, the nucleophilicity *N*′ index affords a low value, *N*′ = 1.89 eV, for this species. A similar behaviour was found for captodative ethylene **4a**_._ For captodative ethylenes **4c**–**d**, both nucleophilicity *N* and *N*′ indices yield similar values.

**Figure 5:**
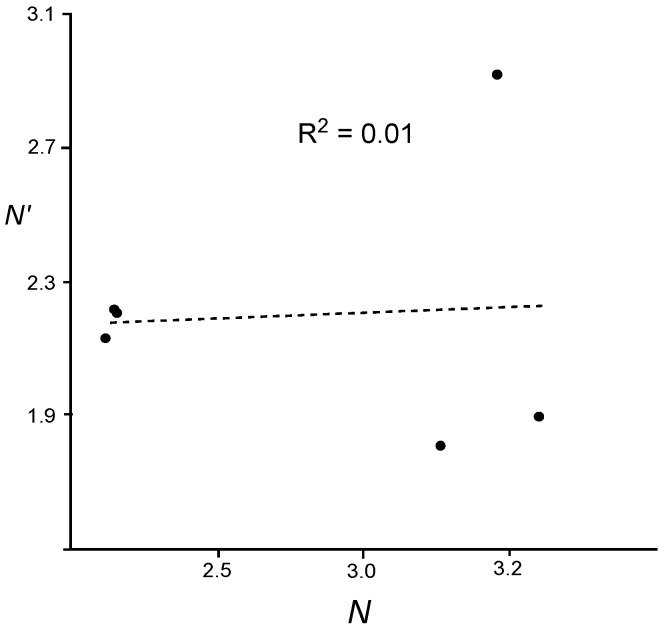
Plot of Roy’s nucleophilicity *N'* index, in eV, *versus* the nucleophilicity *N* index (Equation (27)) for the series of captodative ethylenes **4a**–**f**.

A plot of the nucleophilicity *N*′ index *versus* our nucleophilicity *N* index for the series of captodative ethylenes **4a**–**f** given in Table 4 shows that there is no lineal relation, R^2^ = 0.01 (see Figure 5). The nucleophilicity *N*′ index fails to predict the nucleophilic character of captodative ethylenes **B** presenting both strong electron-drawing and strong electron-donating substituents. In these ambiphilic species, while the electrophilicity ω is well reproduced, the nucleophilicity *N* is underestimated by the *N*′ index.

**Figure 6:**
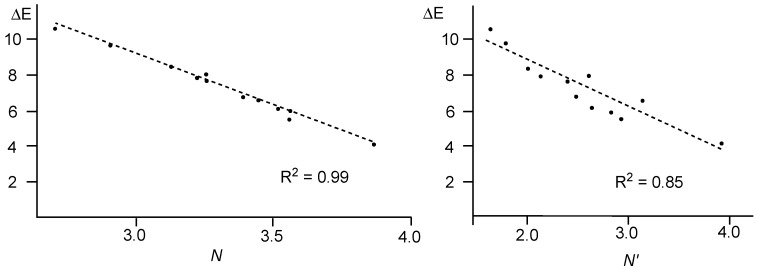
Plot of the activation energies (ΔE^≠^, in kcal/mol) associated with the nucleophilic attack of twelve substituted methoxy bicyclo[2.2.1]hepta-2,5-dienes **D** on 1,1-dicyanoethylene **E** *versus* the nucleophilicity *N* index (R^2^ = 0.99); and the nucleophilicity *N*′ index (R^2^ = 0.85) (see ref. [53]).

A comparative analysis of the nucleophilicity *N* and *N*′ indices of twelve 2-substituted-6-methoxy-bicyclic[2.2.1]hepta-2,5-dienes **D** *versus* the computed activation energy associated with the nucleophilic attack on 1,1-dicyanoethylene **E** (see Scheme 3) showed that the nucleophilicity *N* index yields a better correlation, R^2^ = 0.99, than the *N*′ one, R^2^ = 0.85 (see Figure 6) [53].

These comparative studies asserted that the simple nucleophilicity *N* index derived from E_HOMO_ as an approach of I is an adequate and effective measure of the nucleophilicity of simple and complex organic molecules displaying concurrently electrophilic and nucleophilic behaviours [53].

### 2.6. Local Electrophilicity ω_k_ and Nucleophilicity N_k_ Indices

Similar to the local softness $S_{k}$ [45] and the philicity $\omega_{k}^{\alpha }$ [46] indices, the local electrophilicity $\omega_{k}$ [57] and the local nucleophilicity $N_{k}$ [58] indices, which permit the distribution of the global electrophilicity ω and nucleophilicity *N* indices at the atomic sites k, were defined as:

(29) $$\omega_{k}\;=\;\omega \cdot f_{k}^{+}$$

(30) $$N_{k}\;=\;N\cdot f_{k}^{-}$$

Analysis of the local electrophilicity $\omega_{k}$ and local nucleophilicity $N_{k}$ indices in an organic molecule allows for the characterisation of the most electrophilic and nucleophilic centers in the molecule. A great deal of theoretical work devoted to the study of molecular mechanisms of polar reactions involving non-symmetric reagents has shown that the most favourable reactive channel is that associated with the two-center interaction between the most electrophilic center of a molecule and the most nucleophilic center of the other molecule [15]. Consequently, local electrophilicity $\omega_{k}$ and local nucleophilicity $N_{k}$ indices are able to predict the regio- and chemoselectivity in polar reactions. Numerous experimental and theoretical studies have proven the feasibility of these local descriptors to study regio- and chemoselectivities.

### 2.7. The Parr Functions P(**r**)

**Figure 7:**
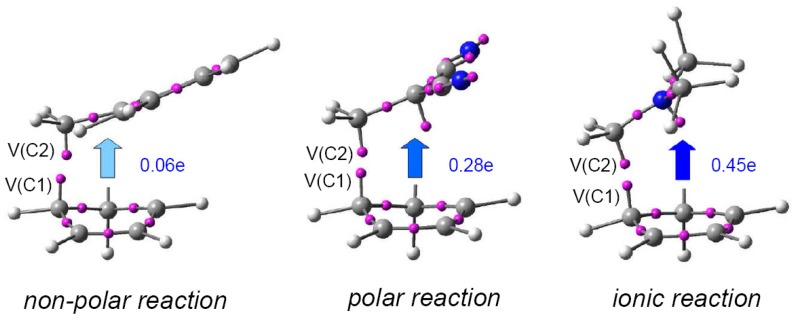
Most relevant data of *pseudodiradical* species involved in the C–C bond formation in non-polar, polar and ionic DA reactions of cyclopentadiene with styrene, dicyanoethylene and *N*-dimethylmethyleneammonium cation. The blue arrows indicate the direction of the GEDT.

ELF topological analysis by way of a large number of studies devoted to the characterisation of the molecular mechanism of different organic reactions involving the participation of C–C double bonds made it possible to establish that in non-polar, polar and ionic reactions the formation of C–C single bonds usually takes place within the short range of 2.0–1.9 Å by a C-to-C coupling of two *pseudoradical* centers [14] generated along the reaction (see Figure 7) [15]. In polar and ionic reactions, the formation of these *pseudoradical* centers is favoured by the GEDT that takes place from the nucleophile to the electrophile. In non-symmetric molecules, a non-symmetric electron density distribution takes place during the GEDT process. Thus, while in the nucleophilic species some atoms lose less electron density, in the electrophilic species some atoms gather more electron density. These relevant atoms correspond to the most nucleophilic and most electrophilic centers of the reactant molecules. Note that the larger the GEDT at the TS, the larger asynchronicity in the C–C single bond formation.

In case an amount equivalent to one electron is transferred, the nucleophile becomes a radical cation, while the electrophile becomes a radical anion. Interestingly, analysis of the atomic spin density (ASD) at the radical cation and the radical anion gives a picture of the distribution of the electron density in the electrophile and the nucleophile when they approach each other along the reaction progress.

Based on these observations, in 2014, Domingo proposed the Parr functions *P*(**r**) [59,60], which are given by the following equations:

(31) $$P^{-}(r)\;=\;\rho_{s}^{\text{rc}}(r)\;\;\;\mathrm{for}\;\mathrm{electrophilic}\;\mathrm{attacks}$$

and

(32) $$P^{+}(r)\;=\;\rho_{s}^{\text{ra}}(r)\;\;\;\mathrm{for}\;\mathrm{nucleophilic}\;\mathrm{attacks}$$

where $\rho_{s}^{rc}$(**r**) is the ASD at the **r** atom of the radical cation of a considered molecule and $\rho_{s}^{ra}$(**r**) is the ASD at the **r** atom of the radical anion. Each ASD gathered at the different atoms of the radical cation and the radical anion of a molecule provides the local nucleophilic $P_{k}^{-}$ and electrophilic $P_{k}^{+}$ Parr functions of the neutral molecule.

**Scheme 4:**
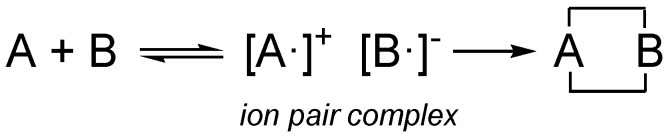
Woodward’s mechanism for Diels-Alder reactions.

Remarkably, prior to the Woodward-Hofmman proposal for the pericyclic mechanism, Woodward proposed in 1942 a mechanism for Diels-Alder reactions in which, before the formation of the two C–C single bonds in the final cycloadducts, an *ion pair complex* is formed by one electron transfer process (see *ion pair complex* [A·]^+^ [B·]^−^ in Scheme 4) [61]. Woodward’s *ion pair complex* mechanism can be seen as an earlier assertion of Domingo’s polar Diels-Alder mechanism [40] in which a zwitterionic transition state is formed resulting from the GEDT. The analysis of the Parr functions can be seen as an analysis of the ASD at the separated [A·]^+^ and [B·]^−^ fragments of Woodward’s *ion pair complex*.

In Table 5, electrophilic $P_{k}^{+}$ and nucleophilic $P_{k}^{-}$ Parr functions, and electrophilic $f_{k}^{+}$ and nucleophilic $f_{k}^{-}$ Parr and Yang [34] (PY) and Yang and Mortier [35] (YM) Fukui functions of four ethylene derivatives are given. A detailed analysis of the three models shows that the YM Fukui functions present some relevant errors. In spite of the fact that the three local functions are normalised, the sum of the YM Fukui functions corresponding to heavy atoms is below 1.0, in discrepancy with the sum of the Parr and the PY Fukui functions, which is closer to 1.0. As can be seen, electrophilic $f_{k}^{+}$ PY and YM Fukui functions give the N and O atoms of nitroso ethylene **5a** as the most electrophilic center, while electrophilic $P_{k}^{+}$ Parr functions correctly predict the C1 carbon as the most electrophilic center of this electron-deficient ethylene. Similarly, for nitroethylene **5b**, electrophilic $f_{k}^{+}$ PY and YM Fukui functions at the N and O atoms are higher than those at the C1 carbon atom, while again, electrophilic $P_{k}^{+}$ Parr functions correctly predict the C1 carbon as the most electrophilic center. For the electron-rich vinyl ether **5d**, nucleophilic $f_{k}^{-}$ YM Fukui functions give the O3 oxygen atom slightly more nucleophilic than the carbon C1, while nucleophilic $P_{k}^{-}$ Parr functions correctly predict the C1 carbon as the most nucleophilic center. For a more comprehensive discussion see reference [59].

Despite the similar electrophilic and nucleophilic local activations given by Parr and PY Fukui functions for the short series of compounds given in Table 5, there are two reasons by which the use of the Parr functions, instead of the PY Fukui ones, is recommended: (i) both functions are conceptually different. The electrophilic $f_{k}^{+}$ PY condensed Fukui functions are obtained from the LUMO electron density (see Equation (14)) as an approximation from the FMO theory. ELF bonding analyses concerning polar reactions involving the participation of C–C double bonds indicate that the C–C single bonds are formed through the C-to-C coupling of two *pseudoradical* centers [15] instead of a HOMO–LUMO donation process as suggested by the FMO theory [62]. Consequently, the LUMO of electrophiles does not participate in the bond formation process; and (ii) Parr functions, obtained by performing simple unrestricted calculations at the radical anion and the radical cation of a molecule, are easier to obtain than PY condensed Fukui functions, which are obtained from the HOMO and LUMO coefficients and the corresponding overlapping integrals using specific programs [63].

With the electrophilic $P_{k}^{+}$ and nucleophilic $P_{k}^{-}$ Parr functions at hand, the local electrophilicity $\omega_{k}$ and the local nucleophilicity $N_{k}$ indices were redefined as follows [59]:

(33) $$\omega_{k}\;=\;\omega \cdot P_{k}^{+}$$

and

(34) $$N_{k}\;=\;N\cdot P_{k}^{-}$$

Therefore, a simple analysis of the Parr functions permits characterising the most electrophilic and the most nucleophilic centers in a molecule. These centers are those with the highest electron density developed along the GEDT involved in polar processes.

### 2.8. Parr Functions and Polar Reactivity

As commented above, electrophilic $P_{k}^{+}$ or nucleophilic $P_{k}^{+}$ Parr functions are able to explain regio- and chemoselectivities in most polar reactions. However, the functionality of Parr functions goes further and, interestingly, within Domingo’s C–C single bond formation model involving C–C double bonds, they are also able to explain the reactivity in polar organic reactions. Hence, the reactions of nitrones is an illustrative example of the applicability of Parr functions in this context. Nitrones are TACs participating in *zwitterionic-type* (*zw-type*) 32CA reactions [64]. Most of the simplest zwitterionic TACs are characterised by a high nucleophilicity *N* index, *N* > 3.0 eV, being classified as strong nucleophiles; consequently, they can react with electron-deficient ethylenes [65]. For these TACs to participate in *zw-type* 32CA reactions towards electron-rich ethylenes, an electrophilic activation of the TAC is demanded. Thus, the simplest nitrone **6a**, ω = 1.06 eV, is a moderate electrophile, and *C*-phenyl-nitrone **6b**, ω = 1.57 eV, is on the borderline of strong electrophiles (see Table 6). Substitution of a hydrogen by a strong electron-withdrawing –NO_2_ group in the phenyl substituent of the nitrone considerably increases the electrophilicity of nitrone **6c** to 3.13 eV. However, in spite of its very high electrophilic activation, the *zw-type* 32CA of **6c** with 2-methylene-1,2-dioxolane, a strong nucleophile, *N* = 3.53 eV**,** presents a high activation energy, 13.2 kcal·mol^−1^, which is similar to that associated with the non-polar 32CA reaction between the simplest nitrone **6a** and ethylene, 13.1 kcal/mol.

**Figure 8:**
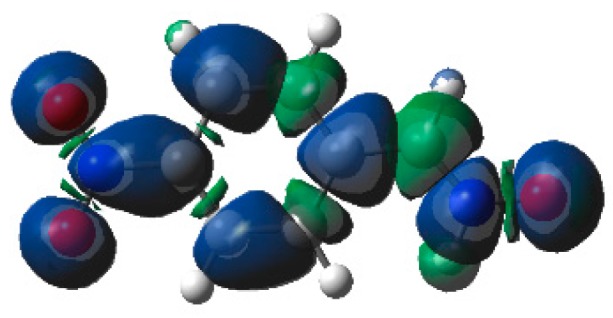
3D representation of the ASD of the radical anion of nitrone **6c**.

The low reactivity of the electrophilically activated nitrone **6c** can be explained by an analysis of the electrophilic $P_{k}^{+}$ Parr functions. As can be seen in Figure 8, electrophilic Parr functions of nitrone **6c** are mainly located at the *p*-nitro-phenyl substituent (see the blue regions of the 3D representations of the ASD of the radical anion of nitrone **6c**). While the nitrone oxygen atom presents a low electrophilic $P_{k}^{+}$Parr function, $P_{O}^{+}$ = 0.18, the nitrone carbon atom is electrophilically deactivated, $P_{C}^{+}$ = −0.07. Consequently, low values of the electrophilic $P_{k}^{+}$ or nucleophilic $P_{k}^{-}$ Parr functions at the atoms involved in the formation of C–C single bonds could account for the low reactivity of electrophilically and nucleophilically activated species in polar reactions.

### 2.9. The Local Reactivity Difference Index R_k_

In order to present simultaneously the electrophilic and/or nucleophilic activation at the different sites of a molecule, in 2012 Chattaraj proposed the local reactivity difference index $R_{k}$, [66] which is able to predict the local electrophilic and/or nucleophilic activation within an organic molecule. The $R_{k}$ index is defined as:

(35) $$\begin{array}{ll} \mathrm{if}\;(1<\omega_{k}/N_{k}<2)\;\mathrm{or}\;(1<N_{k}/\omega_{k}<2 & \;\\ \text{ then }R_{k}\;\approx \;(\omega_{k}\;+\;N_{k})/2\; & \Rightarrow \;\mathrm{ambiphilic}\;(R_{k}\;=\;\pm n.\mathrm{nn})\\ \mathrm{else}\;R_{k}\;\approx \;(\omega_{k}\;+\;N_{k})/2 & \;\\ \text{ where }R_{k}\;>\;0 & \Rightarrow \;\mathrm{electrophilic}\;(R_{k}\;=\;+n.\mathrm{nn})\\ \text{ and }R_{k}\;<\;0 & \Rightarrow \;\mathrm{nucleophilic}\;(R_{k}\;=\;-n.\mathrm{nn})\\ \mathrm{if}\;|R_{k}|\;<\;0.1,\;\mathrm{then}\;R_{k}\;=\;0.0 & \; \end{array}$$

where $\omega_{k}$ and $N_{k}$ are obtained with Equations (33) and (34).

In the $R_{k}$ index, the sign (+, −, ±) indicates the electrophilic and/or nucleophilic character of the center k, while the magnitude n.nn provides a measure of the local activation [66]. For a molecule, the $R_{k}$ molecular map of reactivity (RMMR) represents all local $R_{k}$ indices, giving an illustration of its reactivity in polar processes.

**Figure 9:**
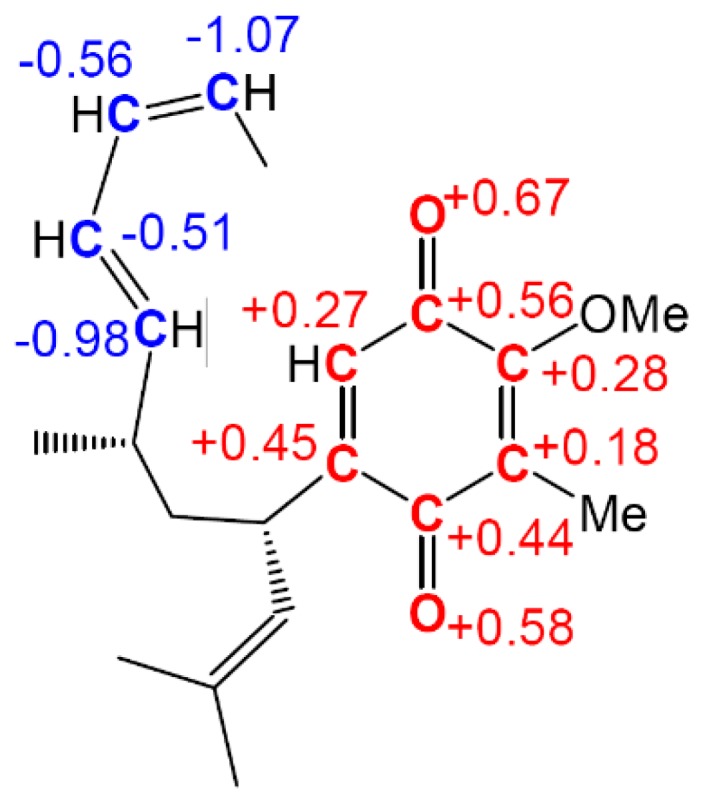
RMMRs (R_k_ molecular maps of reactivity) of a benzoquinone F involved in an intramolecular polar Diels-Alder reaction. $R_{k}\;>\;0$, in red, correspond to electrophilic centers, while $R_{k}\;<\;0$, in blue, correspond to nucleophilic centers.

The R_k_ index has shown to be a powerful tool when studying intramolecular polar reactions, in which electrophilic and nucleophilic centers are located within the same molecule. Figure 9 shows the RMMR of a benzoquinone **F** involved in an intramolecular polar Diels-Alder reaction [67]. Analysis of the RMMR clearly shows that while the butadiene framework of the chain concentrates the nucleophilic reactivity, the benzoquinone core concentrates the electrophilic reactivity.

### 2.10. Electrophilic and Nucleophilic Free Radicals

Non-polar organic reactions involving neutral radical species do not experience any electron density exchange along the reaction. However, the adequate substitution in the radical species may favour the electron density exchange, determining the chemical reactivity of these radical species. Consequently, a set of five DFT reactivity indices, namely, the global electrophilicity $\omega^{ο}$ and nucleophilicity $N^{ο}$ indices, the radical $P_{k}^{ο}$ Parr function and the local electrophilicity $\omega_{k}^{o}$ and nucleophilicity $N_{k}^{o}$ indices, have recently been proposed for the study of the reactivity of free radicals [68].

Using Parr’s definition, the electrophilicity $\omega^{o}$ index of free radicals at their ground state, namely:

(36) $$\omega^{o}\;=\;\frac{\text{µ}{{}^{\circ}}^{2}}{2\eta {}^{\circ}}$$

was introduced, where µ° is the global electronic chemical potential and η° is the global chemical hardness of free radicals. Similar to μ and η, these quantities may be approached as:

(37) $$\mu {}^{\circ}\;\approx \;-\;\frac{\left(\text{I}{}^{\circ}\;+\text{ A}{}^{\circ}\right)}{2}$$

and

(38) η° ≈ - (I° - A°)

where I° and A° correspond to the ionisation potential and the electron affinity of free radicals. Operational formulations for these quantities can be obtained through:

(39) $$\mu {}^{\circ}\;\approx \;\frac{\left(E_{HOMO}^{\alpha ,{}^{\circ}}\;+\;E_{LUMO}^{\beta ,{}^{\circ}}\right)}{2}$$

and

(40) $$\eta {}^{\circ}\;\approx \;-\;(E_{LUMO}^{\beta ,{}^{\circ}}\;-\;E_{HOMO}^{\alpha ,{}^{\circ}})$$

where $E_{HOMO}^{\alpha ,{}^{\circ}}$ and $E_{LUMO}^{\beta ,{}^{\circ}}$ are the one electron energies of the frontier HOMO and LUMO for the electrons in the α and β spin state of the radicals and the relationships *I*° = $-E_{HOMO}^{\alpha ,{}^{\circ}}$ and *A*° = $-E_{LUMO}^{\beta ,{}^{\circ}}$ were used.

On the other hand, and in analogy to our nucleophilicity *N* index, we also introduced the global nucleophilicity *N*° index of free radicals as:

(41) $$N{}^{\circ}\;=\;E_{HOMO}^{\alpha ,{}^{\circ}}(\mathrm{Nucleophile})\;-\;E_{HOMO}^{\alpha ,{}^{\circ}}(\mathrm{DCM})$$

where the dicyanomethyl (DCM) radical is taken as a reference to define a positive scale of global nucleophilicity of radicals.

For the local descriptors, the radical Parr function $P_{k}^{o}$ is defined as:

(42) $$P^{o}(r)\;=\;\rho_{s}(r)$$

where $\rho_{s}$(**r**) is the ASD at the r atom of the neutral radical.

Therefore, the local electrophilicity $\omega_{k}^{o}$ and nucleophilicity $N_{k}^{o}$ indices for free radicals can be easily obtained by the following expressions:

(43) $$\omega_{k}^{o}\;=\;\omega^{o}\cdot P_{k}^{o}$$

(44) $$N_{k}^{o}\;=\;N^{o}\cdot P_{k}^{o}$$

where $\omega^{ο}$ and $N^{ο}$ are obtained from Equations (36) and (41), respectively.

The global radical indices were tested for a series of twenty-three free radicals being electrophilically and/or nucleophilically activated (see Table 7). Thus, analysis of the electrophilicity $\omega^{ο}$ and nucleophilicity $N^{ο}$ indices for the substituted free radicals given in Table 7 clearly shows that they correctly predict their electrophilic and nucleophilic behaviours. Interestingly, these global indices also account for the ambiphilic behaviour of free radicals, such as **7g** and **7h**, having concurrently an electron-withdrawing and electron-releasing substitution.

Analysis of the local electrophilicity $\omega_{k}^{o}$ and nucleophilicity $N_{k}^{o}$ indices for free radicals, together with analysis of the local electrophilicity $\omega_{k}$ and nucleophilicity $N_{k}$ indices for alkenes, allows explaining the regio- and chemoselectivity in radical additions of free radicals to alkenes. ELF topological analysis for the C–C bond formation along the nucleophilic addition of 2-hydroxyprop-2-yl free radical **7u** to electrophilic methyl acrylate evidenced that the new C–C bond is formed through the C-to-C coupling of two radical centers, which were properly characterised through the use of the Parr functions [68]. Besides, radical Parr functions $P_{k}^{o}$ have also shown to be an applicable tool to analyse the most electrophilic and nucleophilic centers in cationic and anionic organic molecules participating in ionic reactions [41,69].

## 3. Conclusions

Reactivity in organic chemistry has been in the spotlight for a long time and is one of the most widely studied and transcendental issues, as its understanding allows determining why and how reactions take place. Since the establishment of DFT as a quantum-mechanical theory founded on electron density, numerous efforts have been devoted to redefine useful concepts already known in organic chemistry and to make them applicable for the semi-quantitative study of reactivity.

In this sense, theoretical reactivity indices defined within the conceptual DFT have become a powerful tool for the semi-quantitative study of organic chemical reactivity. Many reactivity indices have been proposed in the literature, but the purpose of this review has been to collect, and briefly discuss, the most relevant ones, which are those that have proved to give more accurate responses to experimental evidences. Thus, numerous theoretical and experimental studies have shown that global quantities such as the electronic chemical potential μ, the electrophilicity ω and the nucleophilicity *N* indices, and local condensed indices such as the electrophilic $P_{k}^{+}$ and nucleophilic $P_{k}^{-}\;$Parr functions, are powerful tools not only to predict and/or explain reactivity but also provide answers to questions so important to organic chemists, such as regio- and chemoselectivity of organic reactions.

The aim of the present review is to show that conceptual DFT reactivity indices are a highly effective and indispensable tool for analysing reactivity in organic chemistry nowadays and that, remarkably, their calculations can be easily performed without a complex computational demand. This fact constitutes one of their most valuable advantages and makes them accessible to the entire community of organic chemists.

## Figures and Tables

**Table 1:** B3LYP/6-31G(d) electronic chemical potential μ, in eV, of some reagents involved in cycloaddition reactions.

Entry	Molecule	μ
**1a**	(CN)_2_C=C(CN)_2_	−7.04
**1b**	CH_2_=C(CN)_2_	−5.64
**1c**	CH_2_=CHNO_2_	−5.33
**1d**	CH_2_=CHCN	−4.70
**1e**	CH_2_=CHCHO	−4.38
**1f**	CH_2_=CH–CH=CH_2_	−3.46
**1g**	CH_2_=CH_2_	−3.37
**1h**	Cyclopentadiene	−3.01
**1i**	CH_2_=CHOCH_3_	−2.43
**1j**	CH_2_=CHN(CH_3_)_2_	−1.85

**Table 2:** B3LYP/6-31G(d) electrophilicity ω index, in eV, of some common reagents involved in Diels-Alder reactions.

Entry	Molecules	ω
	*Strong electrophiles*	
**2a**	CH_2_=N^+^(CH_3_)_2_	8.25
**2b**	(CN)_2_C=C(CN)_2_	5.96
**2c**	CH_2_=CHCHO:BH_3_	3.20
**2d**	CH_2_=C(CN)_2_	2.82
**2e**	CH_2_=CHNO_2_	2.61
**2f**	CH_2_=CHCHO	1.84
**2g**	CH_2_=CHCN	1.74
**2h**	CH_2_=CHCOCH_3_	1.65
**2i**	CH_2_=CHCO_2_CH_3_	1.51
	*Moderate electrophiles*	
**2j**	CH_2_=CH–CH=CH_2_	1.05
**2k**	CH_2_=CH(CH_3_)–CH=CH_2_	0.94
**2l**	Cycleopentadiene C_5_H_6_	0.83
	*Marginal electrophiles (Nucleophiles)*	
**2m**	CH_3_O–CH=CH–CH=CH_2_	0.77
**2n**	CH_2_=CH_2_	0.73
**2o**	(CH_3_)_2_N–CH=CH–CH=CH_2_	0.57
**2p**	CH≡CH	0.54
**2q**	CH_2_=CHOCH_3_	0.42
**2r**	CH_2_=CHN(CH_3_)_2_	0.27

**Table 3:** B3LYP/6-31G(d) nucleophilicity *N* index, in eV, of some inorganic and organic nucleophilic reagents.

Entry	Molecule	*N*
	*Strong nucleophiles*	
**3a**	CH_2_=CHN(CH_3_)_2_	4.28
**3b**	C_6_H_5_NH_2_	3.72
**3c**	NH_2_NH_2_	3.65
**3d**	CH_2_=C(OCH_3_)_2_	3.51
**3e**	N(CH_3_)_3_	3.48
**3f**	(CH_3_)_2_C=C(CH_3_)_2_	3.35
**3g**	NH(CH_3_)_2_	3.26
**3h**	C_6_H_5_OH	3.16
**3i**	NH_2_CH_3_	3.03
	*Moderate nucleophiles*	
**3j**	CH_2_=CH–CH=CH_2_	2.98
**3k**	C_6_H_5_CH_3_	2.71
**3l**	CH_2_=C(CH_3_)_2_	2.60
**3m**	CH_3_C≡CCH_3_	2.57
**3n**	C_6_H_6_	2.42
**3o**	H_2_O_2_	2.41
**3p**	C_6_H_5_COCH_3_	2.39
**3q**	CH_2_=CHCH_3_	2.32
**3r**	NH_3_	2.25
**3s**	NH_2_OH	2.19
**3t**	C_6_H_5_CHO	2.17
**3u**	C_6_H_5_CO_2_H	2.03
	*Marginal nucleophiles*	
**3v**	CH_3_OH	1.92
**3x**	CH_2_=CH_2_	1.86
**3y**	C_6_H_5_NO_2_	1.53
**3z**	H_2_O	1.20

**Table 4:** B3LYP/6-31G(d) electrophilicity (ω) and nucleophilicity (*N*) and Roy’s nucleophilicity (*N*′) values of some captodative ethylenes **4a**–**f** (see Scheme 2).

Entry	A	D	ω	*N*	*N*′
**4a**	–NO	–PhOCH_3_	3.00	3.26	1.81
**4b**	–NO	–N(CH_3_)_2_	2.52	3.29	1.89
**4c**	–COMe	–OCOPh	1.85	2.12	2.13
**4d**	–COOMe	–OCOPh	1.73	2.15	2.20
**4e**	–COMe	–OCOMe	1.72	2.15	2.21
**4f**	–CN	–N(CH_3_)_2_	1.08	3.53	2.92

**Table 5:**
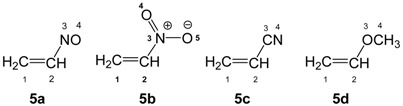
Electrophilic $P_{k}^{+}$ and nucleophilic $P_{k}^{-}$ Parr functions, and electrophilic $f_{k}^{+}$ and nucleophilic $f_{k}^{-}$ PY and YM Fukui functions of four common ethylene derivatives.

Entry					PY		YM	
			$P_{k}^{+}$	$P_{k}^{-}$	$f_{k}^{+}$	$f_{k}^{-}$	$f_{k}^{+}$	$f_{k}^{-}$
**5a**	1	C	0.41	−0.09	0.29	0.01	0.11	0.10
	2	C	−0.06	0.08	0.04	0.09	−0.01	0.01
	3	N	0.31	0.30	0.35	0.34	0.21	0.20
	4	O	0.38	0.68	0.33	0.54	0.26	0.34
**5b**	1	C	0.44	−0.01	0.28	0.01	0.11	0.07
	2	C	0.01	0.01	0.08	0.01	0.03	0.02
	3	N	0.23	−0.05	0.23	0.02	0.07	0.04
	4	O	0.22	0.83	0.22	0.52	0.22	0.37
	5	O	0.16	0.20	0.19	0.43	0.20	0.21
**5c**	1	C	0.63	0.44	0.47	0.37	0.13	0.14
	2	C	0.20	0.27	0.27	0.27	0.11	0.11
	3	C	0.03	−0.07	0.09	0.09	0.14	0.18
	4	N	0.22	0.41	0.18	0.28	0.17	0.19
**5d**	1	C	0.48	0.58	0.44	0.47	0.13	0.16
	2	C	0.50	0.07	0.46	0.20	0.16	0.06
	3	O	0.00	0.36	0.05	0.28	0.03	0.17

**Table 6:** B3LYP/6-31G(d) electrophilicity ω and nucleophilicity *N* indices, in eV, of nitrones **6a**–**c**.

Entry	Nitrone	ω	*N*
**6a**	CH_2_NH^+^O^–^	1.06	2.92
**6b**	Ph–CHNH^+^O^–^	1.57	3.51
**6c**	*p*-NO_2_–Ph–CHNH^+^O^–^	3.13	2.77

**Table 7:**
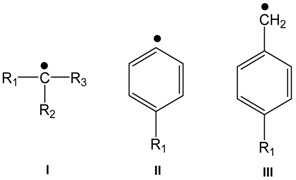
B3LYP/6-31G(d) electronic chemical potential, ${\text{µ}}^{ο}$, chemical hardness, $\eta^{ο}$ , global electrophilicity, $\omega^{ο}$ , and global nucleophilicity, $N^{ο}$ , in eV, for a series of thirty-two free radicals ordered by decreasing electrophilicity.

Entry	Type	R_1_	R_2_	R_3_	$\mu^{ο}$	$\eta^{ο}$	$\omega^{ο}$	$N^{ο}$
**7a**	I	CN	CN	H	−6.35	3.08	6.54	0.00
**7b**	I	COMe	CO_2_Me		−5.67	3.10	5.18	0.67
**7c**	III	NO2			−4.58	2.46	4.28	2.08
**7d**	I	CN	H	H	−5.29	3.70	3.79	0.75
**7e**	III	CN			−4.30	2.57	3.61	2.31
**7f**	III	CN			−5.00	4.52	2.77	0.62
**7g**	I	CN	OH		−4.12	3.28	2.59	2.13
**7h**	I	CN	OMe	H	−3.99	3.16	2.53	2.32
**7i**	III	H			−3.47	2.80	2.15	3.02
**7j**	II	OMe			−4.33	4.51	2.08	1.31
**7k**	II	H			−4.24	4.45	2.02	1.42
**7l**	II	Me			−4.19	4.46	1.97	1.47
**7m**	III	OMe			−3.07	2.64	1.79	3.50
**7n**	III	NH_2_			−2.82	2.54	1.56	3.80
**7o**	I	H	H	H	−3.70	4.80	1.43	1.79
**7p**	I	Cl	Me	Me	−3.23	3.85	1.36	2.73
**7k**	I	Me	H	H	−3.13	4.42	1.10	2.55
**7r**	I	Me	Me	H	−2.78	4.12	0.94	3.05
**7s**	I	Me	Me	Me	−2.57	3.88	0.85	3.38
**7t**	I	OMe	H	H	−2.24	3.96	0.63	3.67
**7u**	I	OH	Me	Me	−2.11	3.84	0.58	3.86
**7v**	I	NH_2_	H	H	−1.93	3.92	0.48	4.00
**7z**	I	OMe	OMe	H	−1.35	3.61	0.25	4.74
